# Characterization and quantification of dynamic eccentric regurgitation of the left atrioventricular valve after atrioventricular septal defect correction with 4D Flow cardiovascular magnetic resonance and retrospective valve tracking

**DOI:** 10.1186/s12968-015-0122-4

**Published:** 2015-02-19

**Authors:** Emmeline E Calkoen, Jos JM Westenberg, Lucia JM Kroft, Nico A Blom, Mark G Hazekamp, Marry E Rijlaarsdam, Monique RM Jongbloed, Albert de Roos, Arno AW Roest

**Affiliations:** Division of Paediatric Cardiology, Department of Paediatrics, Leiden, The Netherlands; Department of Anatomy and Embryology, Leiden, The Netherlands; Department of Radiology, Leiden, The Netherlands; Department of Thoracic surgery, Leiden, The Netherlands; Department of Pediatric Cardiology, Leiden University Medical Center, J6 Albinusdreef 2, Leiden, ZA 2333 The Netherlands

**Keywords:** 4DFlow, Mitral valve regurgitation, Atrioventricular septal defect, Cardiovascular magnetic resonance

## Abstract

**Background:**

To characterize and directly quantify regurgitant jets of left atrioventricular valve (LAVV) in patients with corrected atrioventricular septal defect (AVSD) by four-dimensional (4D)Flow Cardiovascular Magnetic Resonance (CMR), streamline visualization and retrospective valve tracking.

**Methods:**

Medical ethical committee approval and informed consent from all patients or their parents were obtained. In 32 corrected AVSD patients (age 26 ± 12 years), echocardiography and whole-heart 4DFlow CMR were performed. Using streamline visualization on 2- and 4-chamber views, the angle between regurgitation and annulus was followed throughout systole. On through-plane velocity-encoded images reformatted perpendicular to the regurgitation jet the cross-sectional jet circularity index was assessed and regurgitant volume and fraction were calculated. Correlation and agreement between different techniques was performed with Pearson’s r and Spearman’s rho correlation and Bland-Altman analysis.

**Results:**

In 8 patients, multiple regurgitant jets over the LAVV were identified. Median variation in regurgitant jet angle within patients was 36°(IQR 18–64°) on the 2-chamber and 30°(IQR 20–40°) on the 4-chamber. Regurgitant jets had a circularity index of 0.61 ± 0.16. Quantification of the regurgitation volume was feasible with 4DFlow CMR with excellent correlation between LAVV effective forward flow and aortic flow (r = 0.97, p < 0.001) for internal validation and moderate correlation with planimetry derived regurgitant volume (r = 0.65, p < 0.001) and echocardiographic grading (rho = 0.51, p = 0.003).

**Conclusions:**

4DFlow CMR with streamline visualization revealed multiple, dynamic and eccentric regurgitant jets with non-circular cross-sectional shape in patients after AVSD correction. 4DFlow with retrospective valve tracking allows direct and accurate quantification of the regurgitation of these complex jets.

## Background

After correction of an atrioventricular septal defect (AVSD), regurgitation of the left atrioventricular valve (LAVV, i.e. the left part of the common atrioventricular valve after correction, connecting the left atrium with left ventricle) is common. Up to 11% of the patients with corrected AVSD require surgery of the LAVV during follow-up [1], and according to the grown-up congenital heart disease (GUCH) guidelines of the European Society Cardiology (ESC) surgical correction of LAVV regurgitation should be considered in asymptomatic patients with moderate to severe LAVV [2]. Therefore, reliable quantification of LAVV regurgitation is important for clinical decision making.

Echocardiography is most commonly used to evaluate mitral valve or LAVV regurgitation, but echocardiographic quantification of the regurgitation is based on several assumption, such as a cross-sectional circular shape of the jet and a static occurrence and position of the jet throughout systole. In adult patients with mitral regurgitation of diverse origin, poor inter-observer agreement has been described for echocardiographic grading of mitral valve regurgitation using proximal isovelocity surface area (PISA) and vena contracta methods in cases of eccentric regurgitant jets, with non-circular cross-sectional shape or for non-pansystolic regurgitation [3,4]. Also in patients with corrected AVSD, quantification of LAVV regurgitation with echocardiography has shown poor inter-observer agreement, possibly caused by the eccentric and multiple, non-circular characteristics of the jets [5]. Consequently, in patients after AVSD correction or patients with complex mitral valve regurgitation, no accurate reference method is available to assess LAVV/mitral valve regurgitant volume and fraction; for such cases, a combination of approaches is suggested [6,7].

Four-dimensional (4D) Flow Cardiovascular Magnetic Resonance (CMR) allows visualization and reliable, validated and direct quantification of mitral valve flow using retrospective valve tracking perpendicular to inflow and regurgitant jet with the opportunity to perform internal validation [8,9]. However, this approach has not been applied in patients after AVSD correction.

We hypothesize that the dynamics and shape of the regurgitant jets after AVSD correction are not in accordance with the assumptions on which the currently available direct quantification techniques are based. Furthermore, we hypothesize that 4DFlow CMR with retrospective valve tracking allows direct and accurate quantification of LAVV regurgitation by tracing the regurgitant jet(s) throughout systole with good internal validation of LAVV flow with aortic flow. Therefore, the purpose of the current study was to characterize and directly quantify regurgitant jets of the LAVV in patients with corrected AVSD by 4DFlow CMR, streamline visualization and retrospective valve tracking.

## Methods

### Study population

The study protocol was approved by the The medical ethical committee of the Leiden University Medical Center and informed consent was obtained from participants or their parents. Inclusion criteria were a history of AVSD correction and compatibility for CMR (age above 8 years, no-pacemaker dependency, non-Down syndrome). Thirty-four patients were prospectively enrolled between October 2012 and October 2013 from an available surgical database [1]. Twenty-five out of the thirty-four cases have been previously reported [10]. This prior article focussed on the optimal CMR quantification of diastolic left ventricular inflow, whereas in the current manuscript we report on regurgitation during systole. Two cases were excluded because of a history of LAVV replacement. To assess grading of LAVV regurgitation, patients underwent echocardiography and 4DFlow CMR. Transthoracic echocardiography images were acquired using a commercially available system equipped with a 3.5 MHz transducer (Vivid 9, GE-Vingmed Ultrasound, Horton, Norway). A senior paediatric cardiologist (MR) with over 25 years of experience in echocardiography of congenital heart defects, visually classified/graded the regurgitation as none/trace, mild, moderate or severe, blinded for the 4DFlow results [5].

### Cardiovascular magnetic resonance

A 3 T CMR system (Ingenia, Philips Medical Systems, the Netherlands) with maximal amplitude of 45 mT/m for each axis and a slew rate of 200 T/m/sec, with a combination of FlexCoverage Posterior coil in the table top with a dStream Torso coil, providing up to 32 coil elements for signal reception was used. Whole-heart 4DFlow was performed with velocity-encoding of 150 cm/s in all three directions, spatial resolution 2.3 × 2.3 × 3.0 mm^3^, flip angle 10°, echo time (TE) 3.2 ms, repetition time (TR) 7.7 ms, true temporal resolution 31 ms, SENSE factor 2 in anterior-posterior direction and Echo Planar Imaging with a factor 5. No segmented k-space acquisition was used. Mean acquisition time of the 4DFlow scan was 8 minutes (range 5–11 minutes). Commercially-available concomitant gradient correction and local phase correction filter were applied, from the software available on CMR system (Ingenia 3 T with Software Stream 4.1.3.0). Just prior to the 4DFlow acquisition, in the majority (26 out of 32) of patients Gadolinium contrast agent (0.015 mmol/kilogram body weight, Dotarem®, Guerbet, Aulnay-sous-Bois, France) was administrated, for other clinical evaluations. Furthermore prior to contrast administration, cine 2D left 2-chamber, 4-chamber, coronal and sagittal aorta views and a cine muli-2D short-axis stack of slices were acquired, using steady-state free-precession sequences with TE/TR 1.5/3.0, 350 mm field-of-view, 45° flip angle, acquisition resolution 1.0 × 1.0 × 8.0 mm^3^. Retrospective gating was used with 30 phases reconstructed to represent one cardiac cycle. Free breathing was allowed without using motion suppression, three signal averages were taken to suppress effects of breathing motion.

### CMR analysis

Image analysis was performed by one observer (EC) with two years of experience in CMR and verified by two observers (JW and LK) with over 15 years of experience in CMR. Image analysis was done using in-house developed Mass software (Leiden).

Left ventricular end-diastolic volume (LVEDV) and end-systolic volume (LVESV) were calculated by planimetry: the endocardial border was manually traced at end-diastole and end-systole in short-axis slices, the enclosed areas were calculated, multiplied by slice thickness and summed over all slices. Ejection fraction (EF) was calculated as EF = (LVEDV ‐ LVESV)/LVEDV. The maximum left atrial volume (LAV) was calculated according to the biplane area-length from the 2- and 4-chamber view using the formula (8/3π × Area(4 ‐ chamber) × Area(2 ‐ chamber))/(shortest atrial length). Atrial and ventricular volume were indexed for body surface area (BSA) according to Du Bois formula [11].

**Figure 1:**
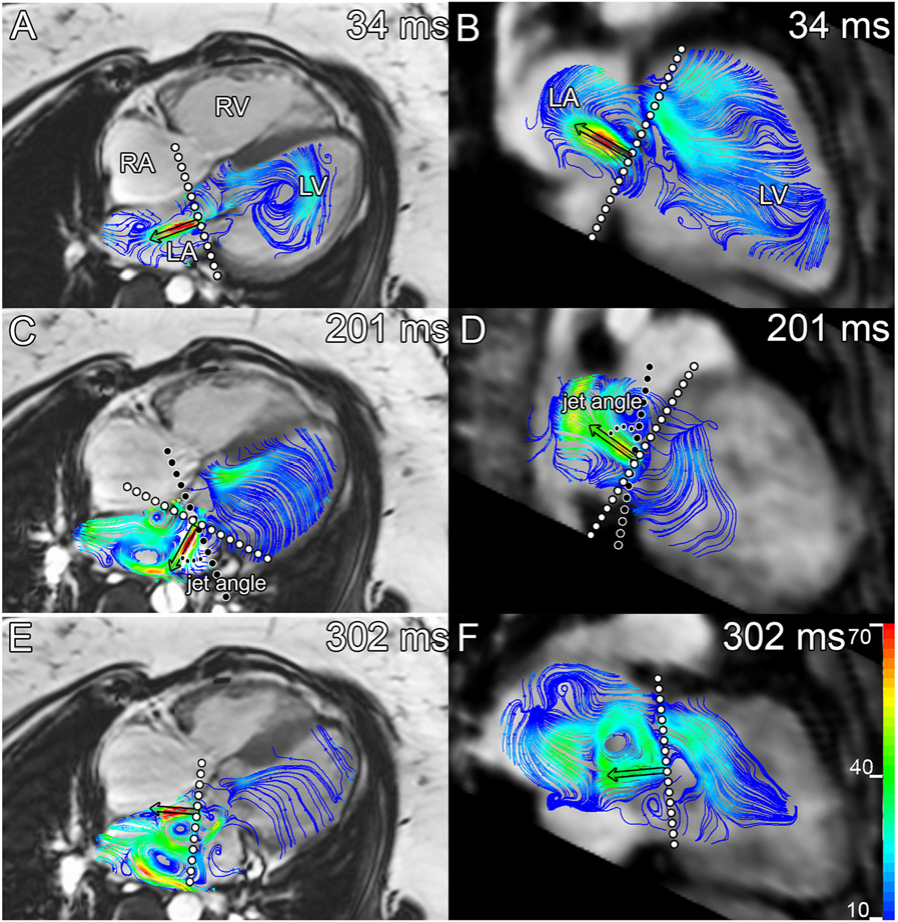
**Multiple dynamic eccentric regurgitant jets.** In one subjects in 4-chamber (A, C, E) and 2-chamber (B, D, F) view during early- (34 ms) (A,B), mid- (201 ms) (C,D) and late (302 ms) (E,F) systole (total RR 984 ms). The jet starts nearly perpendicular to the annulus **(A, B)**, but changes to a more lateral **(C)** and anterior direction **(D)**. A second jet was observed in late systole **(E)**. Black dots show the annulus plane and the white dots the plane perpendicular to the jet. In plane **(C, D)**, the angle measurements are illustrated between annulus and regurgitant jet.

To assess the dynamics of the LAVV regurgitant jet, the jet direction was visualized using streamlines [12], in two orthogonal stacks of parallel cine multiplanar reformatting planes (MPRs) in 2- and 4-chamber orientation, constructed from the magnitude gradient-echo images. At each phase during systole, the MPR with the largest regurgitant jet projection was used to measure the angle between the jet and the valve annulus (Figure 1).

**Figure 2:**
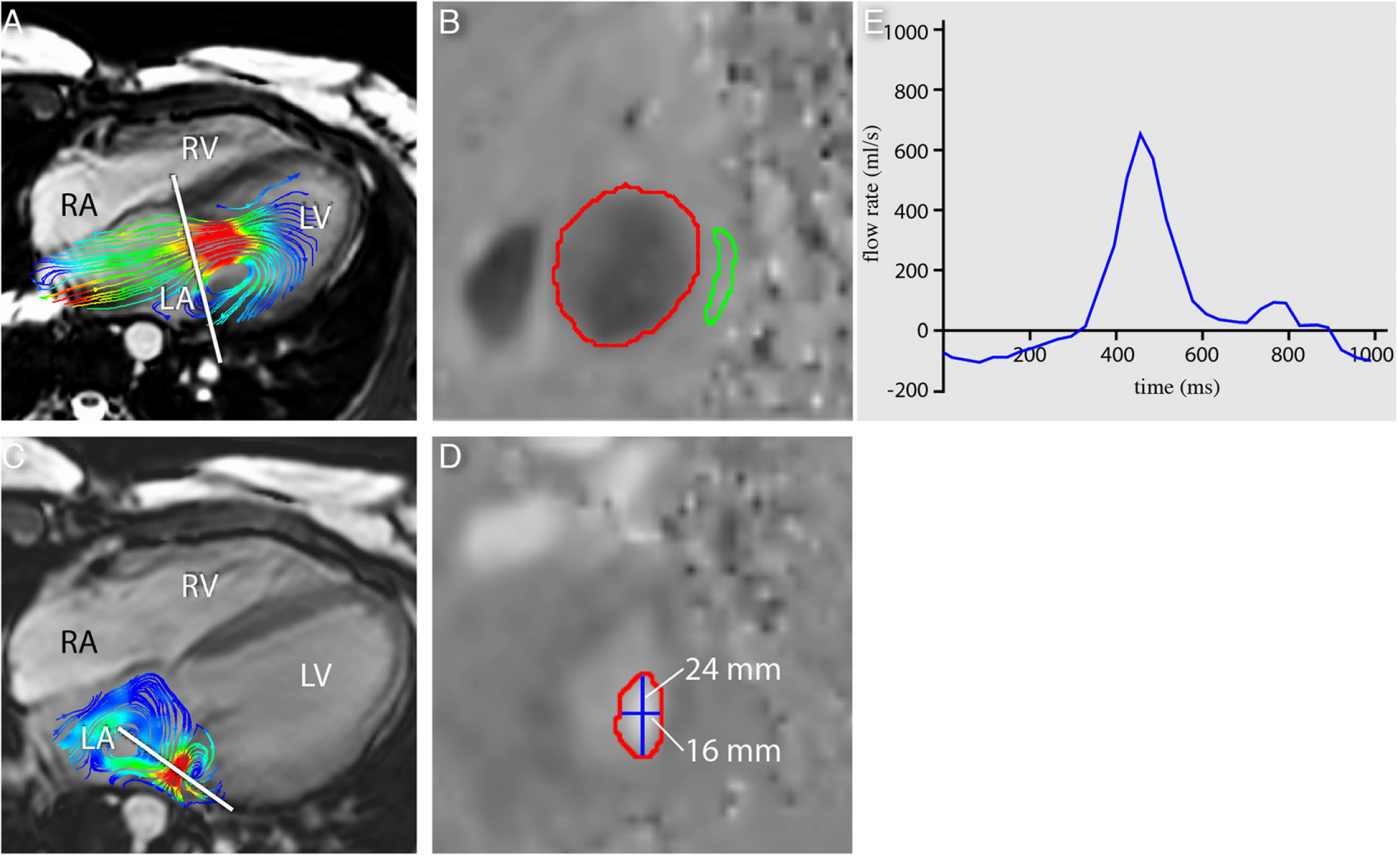
**Example of flow quantification.** Retrospective valve tracking in a patient with the reformatting plane (white line) adjusted to the inflow during diastole **(A)** and perpendicular to the regurgitant jet during systole **(C)** with through-plane velocity of inflow (**B** red contour indicates LAVV flow, green contour traces a region within the free wall of the left ventricle for background correction) and the non-circular regurgitant jet (CI = 0.67) **(D)**. In **(E)** the flow curve.

Prior to velocity mapping, the source velocity images were checked for aliasing artefacts. In case aliasing occurred in the region of interest, phase unwrapping was performed in the source images. Retrospective valve tracking was performed to assess trans-LAVV and aortic flow volumes as described previously [9]. In the presence of LAVV regurgitation, the reformatting plane was positioned perpendicular to the regurgitant jet, 1–2 cm proximal to the valve throughout systole, to avoid sampling in an area with phase dispersion due to turbulent flow at the orifice of the regurgitation (Figure 2) [13]. In case of multiple regurgitant jets with different directions, a separate reformatting planes was constructed for each jet. The total regurgitant volume was determined by summing the regurgitant volume of each of the jets. To assess the shape of the regurgitant jet, the circularity index of the jet at the phase of maximum regurgitant flow was calculated as the shortest diameter/longest cross-sectional diameter (Figure 2). To determine diastolic inflow volume during diastole, the MPR plane was positioned perpendicular to the inflow direction (Figure 2) and tracking the valve. Transvalvular flow velocity was calculated by subtraction of myocardial velocity in through-plane direction from the mean velocity measured over the inflow area [14]. Transvalvular flow volume was subsequently calculated by integrating the transvalvular velocity over the inflow area (i.e., the flow rate) and then integrating over the cardiac cycle (Figure 2E). In case of aliasing, phase unwrapping of the original data set was performed in the source data using in-house developed software. The phase unwrapping algorithm uses the linear relation between image grey value and the velocity scale between -Venc and + Venc to recalculate velocity values exceeding this scale in wrapped areas [15]. Regurgitant fraction was calculated as the ratio between regurgitant volume and total flow volume over LAVV × 100%. For internal validation, the effective forward flow over the LAVV was compared with the aortic flow volume. Aortic flow volume was assessed from a similar retrospective valve tracking procedure at the aortic valve, obtained from the same 4DFlow acquisition.

For comparison of 4DFlow derived regurgitation volume with a conventional CMR quantification technique, regurgitation volume was also quantified as the difference between planimetry-derived stroke volume (LVEDV-LVESV), based on short-axis images minus aortic flow, derived from the 4DFlow acquisition.

### Statistical analysis

Variables were tested for normal distribution using the Shapiro-Wilk test. Continuous variables are expressed as mean ± standard deviation (SD) and as median with inter-quartile range (IQR) where appropriate. Linear regression analysis and Bland-Altman analysis [16] were performed to assess agreement between LAVV effective forward flow and aortic flow. Correlation between left atrial and ventricular volume with regurgitation volume was assessed with linear regression analysis (Pearson’s r). Spearman’s regression analysis (rho) were used to assess correlation between regurgitant flow volume measured on 4DFlow CMR and visual grading of regurgitation. Data analysis was performed using SPSS Statistics (version 20.0 IBM SPSS, Chicago, Illinois).

## Results

Patient characteristics are presented in Table 1. Twenty-one (66%) patients underwent correction of a partial AVSD and 11 (34%) patients underwent correction of a complete AVSD at a median age of 52 (IQR 8–100) months. Patients were examined 20±9 years after correction. 4DFlow CMR data of all 32 patients were visually inspected to be of sufficient quality for image analysis and streamline visualization and trans-valvular flow quantification was possible. In one patient aliasing occurred in the regurgitation jet and phase unwrapping was performed.

**Table 1:** **Patient characteristics**

	**Patients**
N	32
Male (N, %)	9 (28%)
Age (years)	26 ± 12
BSA (m^2^)	1.7 ± 0.3
BMI	23 (IQR 19–29)
LVEDV / BSA (ml/m^2^)	91 ± 15
LAESV / BSA (ml/m^2^)	45 ± 11
EF LV (%)	55 ± 5
NYHA class	30 (94%) class I, 2 (6%) class II
Type AVSD	21 (66%) partial, 11 (34%) complete
Age surgery (months)	52 (IQR 8–100)
Time after surgery (years)	20 ± 9

Data are presented as mean ± standard deviation or median with interquartile range where appropriate. *Indicates p < 0.05.

BSA = Body Surface Area, BMI = Body mass index, LVEDV = Left ventricular end diastolic volume, LAESV = Left atrial end systolic volume, EF = Ejection Fraction, LV = Left ventricle, NYHA class = New York Heart Association classification, AVSD = Atrioventricular septal defect.

Of 32 included patients, 2 patients underwent re-operation of the LAVV due to regurgitation. Mean left ventricular EF was 55±5% and 30 (94%) patients were categorized NYHA class I and two (6%) patients NYHA class II. Using echocardiography, LAVV regurgitation was classified based on visual grading as none to trace in 3 patients, mild in 15 patients and moderate in 14 patients.

### Dynamics of LAVV regurgitation

**Figure 3:**
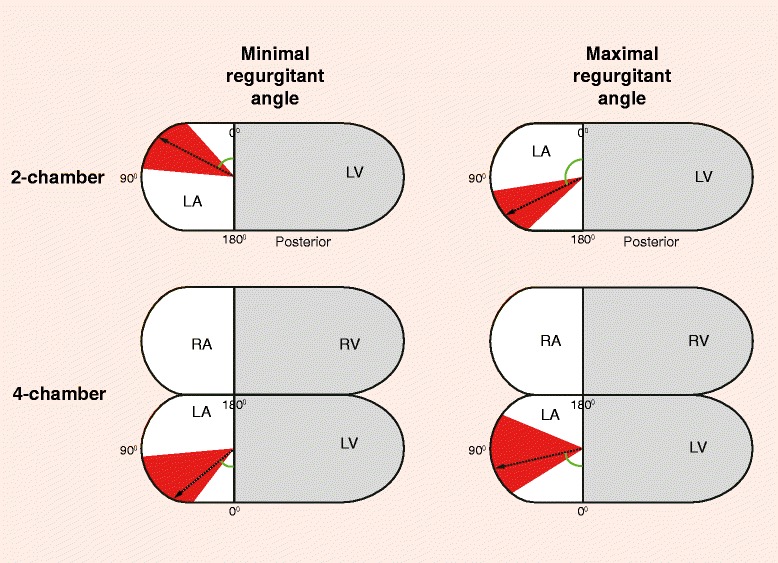
**Schematic representation of the regurgitant jet angle in 2- and 4-chamber view.** The median (black arrow) and interquartile range (red) of the minimal and maximal regurgitant angles are presented. LA = left atrium, LV = left ventricle, RA = right atrium, RV = right ventricle.

In 26 patients, the LAVV regurgitant jet(s) were visualized with streamlines. The six patients (including the 3 patients with none to trace LAVV regurgitation) in whom the LAVV regurgitant could not be visualized with streamlines were quantified with a low regurgitation volume (1–4ml). In 8 (31%) patients, multiple LAVV regurgitant jets were observed (Figure 1). All regurgitant jets were dynamic with changes in the jet angle during systole. Minimal and maximal angle of the jet showed large variation between patients (Table 2 and Figure 3), with a lateral orientation of the jet on the 4-chamber view and various orientation on the 2-chamber view. The median difference between minimal and maximal LAVV regurgitant jet angle within patients was 36° (IQR 18–64°) on the 2-chamber and 30° (IQR 20–40°) on the 4-chamber. Cross-sectional jets areas were non-circular (Figure 2D), as evidenced by a circularity index of 0.61±0.16. No differences in jet dynamics were observed between the patients with partial AVSD and complete AVSD.

**Table 2:** **Regurgitant jet angle (degrees) on 2- and 4-chamber view**

	**Minimal angle**	**Maximal angle**	**Angle difference**
4-chamber view	50° (IQR 37–85°)	77° (IQR 58–112°)	30° (IQR 20–40°)
2-chamber view	63° (IQR 42–90°)	116° (IQR 81–133°)	36° (IQR 18–64°)

Data are presented as median with interquartile range.

### Quantification of LAVV regurgitation

**Figure 4:**
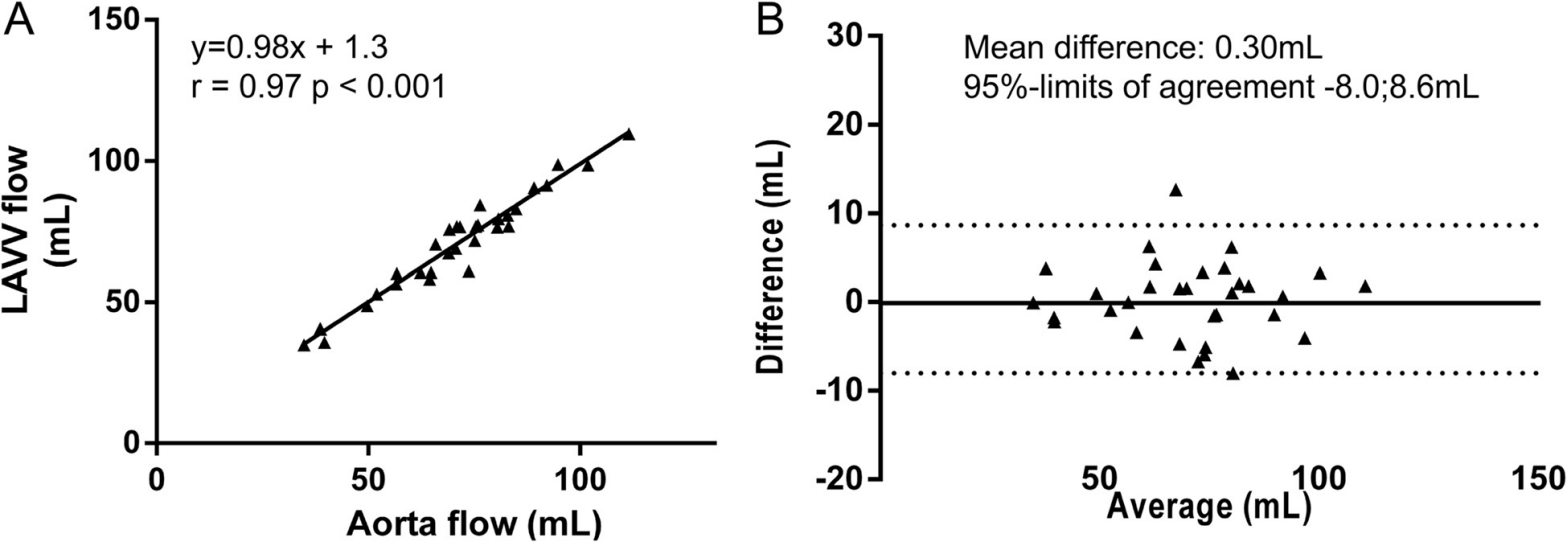
**Correlation (A) and agreement (B) between aortic flow and net LAVV flow.**

**Figure 5:**
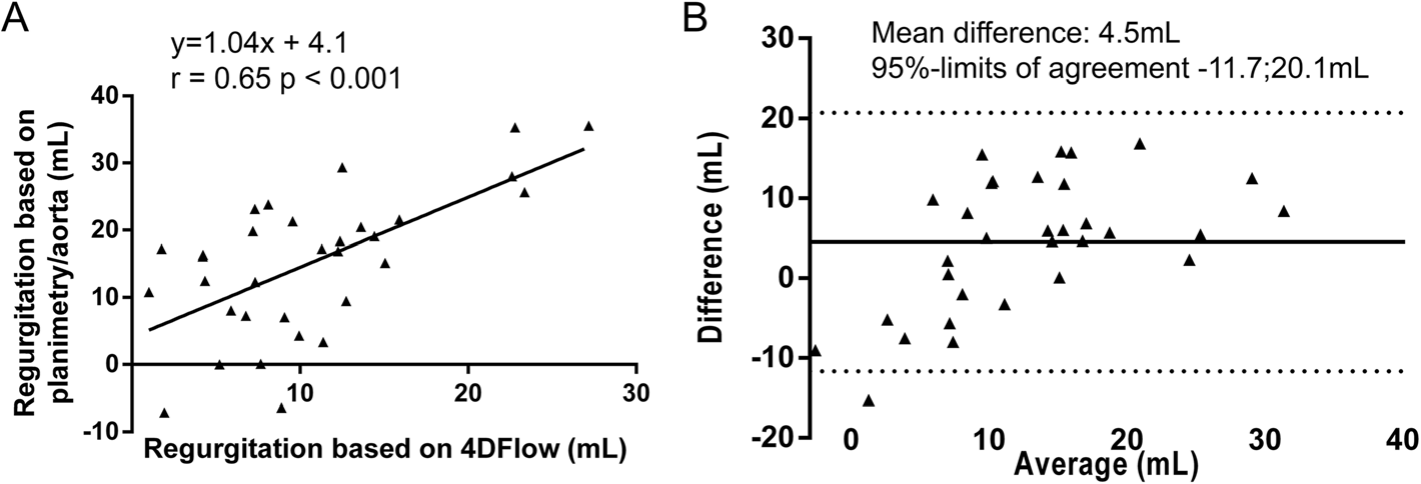
**Correlation (A) and agreement (B) between regurgitant volume based on planimetry minus aortic flow and regurgitant volume based on direct quantification from 4DFlow data.**

**Figure 6:**
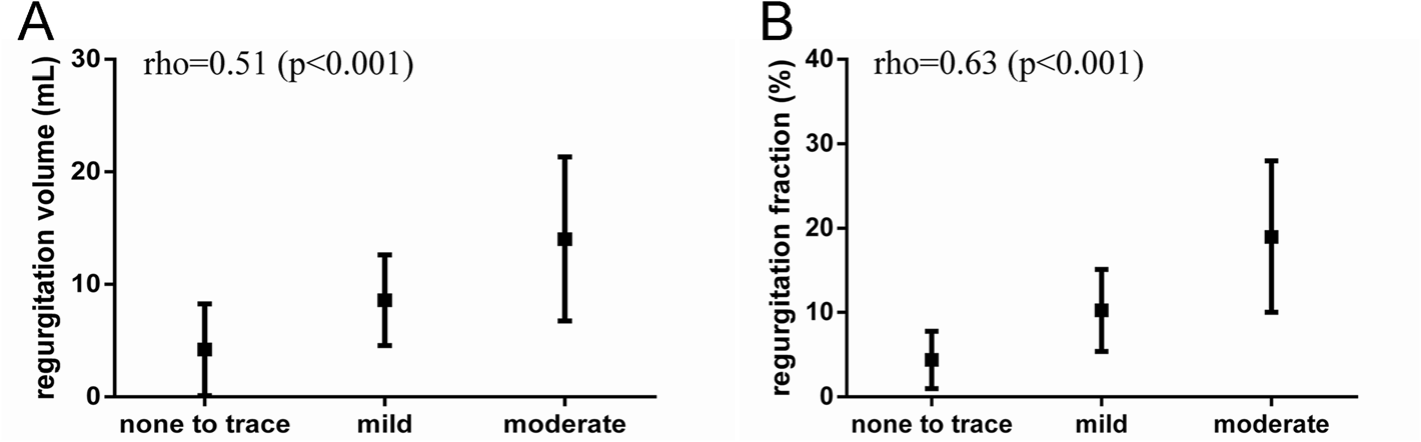
**Correlation between regurgitation classification based on visual echocardiography and CMR quantification.** Mean and standard deviation of the quantitative measurements per visually scored regurgitation grade are given: **(A)**: regurgitation volume based on 4DFlow CMR and **(B)**: regurgitation fraction based on 4D flow CMR.

Using 4DFlow CMR with retrospective plane tracking and streamline visualization, mean LAVV regurgitant volume was 11 ± 6 ml and mean regurgitant fraction was 14 ± 8%. Excellent correlation (Pearson’s r = 0.97, p < 0.001) and agreement (mean difference 0.3 ± 4.0 ml, p = 0.69, 95%-limit of agreement-8.0;8.6 ml) was observed between aortic flow and LAVV effective forward flow (Figure 4). Correlation of the regurgitation volume derived from direct 4DFlow CMR with the combined method of planimetry - aortic flow method was Pearson’s r = 0.65 (p < 0.001) (Figure 5). Correlation between planimetry (stroke volume) and aortic flow plus regurgitation fraction was Pearson’s r = 0.90. Regurgitant flow volume correlated with LAV relative to BSA (Pearson’s r = 0.53, p = 0.002) and LVEDV relative to BSA (Pearson’s r = 0.44, p = 0.016) in 30 patients with a corrected AVSD who did not underwent re-operation. Correlation between visual grading and 4DFlow CMR assessment of 4DFlow regurgitation volume was rho = 0.51 (p = 0.003) and of regurgitation fraction rho = 0.63 (p <0.001) (Figure 6A,B).

## Discussion

The key findings of current study were as follows: First, in patients with corrected AVSD, LAVV regurgitant jets are dynamic, eccentric and have a non-circular cross-sectional shape. Second, it is feasible to directly quantify LAVV regurgitation using 4DFlow CMR and retrospective valve tracking with excellent internal validation with aortic flow and this approach showed moderate correlation with the planimetry-based CMR approach.

In patients after AVSD correction, recurrent regurgitation of the LAVV is common and associated with leaflet prolapse, rest cleft, large annular area and a different position of the papillary muscle [1,17]. Reoperation incidence due to LAVV regurgitation is as high as 11%. The ESC GUCH guidelines recommend surgical correction in case of moderate or severe LAVV regurgitation in symptomatic as well as in asymptomatic patients with signs of LV volume overload. Recommendations are however largely based on level C evidence (consensus of opinion of the experts and/or small studies, retrospective studies, registries). Clear indications for and timing of intervention remain debatable in this patient group, in part based on difficulties in the reliable assessment of the amount of LAVV regurgitation [18].

Little is known about the natural history or predictive value of chronic LAVV regurgitation in patients with corrected AVSD. Adult patients with asymptomatic chronic organic mitral valve regurgitation show a high likelihood to develop cardiac symptoms and high incidence of mortality [19-22]. Chronic mitral valve regurgitation can remain asymptomatic for years, due to the increase in left atrial and ventricular volume [23], which was also found in our study. Due to this compensatory mechanism ejection fraction can remain normal despite myocardial dysfunction [24]. However, left atrial enlargement is a predictor of mortality and atrial fibrillation in patients with chronic mitral valve regurgitation due to flail leaflets or mitral valve prolapse and should be considered in clinical decision making for mitral valve correction [25-27]. Prolonged volume overload eventually results in cardiac hypertrophy and contractile dysfunction, with impaired output, increased end systolic volume and pulmonary congestion. Because of the poor prognosis of organic mitral valve regurgitation even in asymptomatic patients, early correction of organic mitral valve regurgitation should be considered according to adult guidelines [23].

Currently data on long-term outcome of patients with LAVV regurgitation after AVSD correction is lacking. This is complicated by the lack of a gold standard to quantify LAVV regurgitation in these patients. Furthermore, not all suggested quantification techniques have been evaluated in children with LAVV regurgitation after AVSD correction and no established cut-off values are available for LAVV/mitral valve regurgitation severity in children [18]. The observed eccentric, dynamic, multiple, non-circular jets in the current study can explain the reported low inter-observer agreement found in previous studies evaluating LAVV regurgitation after AVSD repair [5], because quantitative echocardiography techniques are based on several assumptions. Eccentric regurgitant jets, in contrast to central jets, are in close contact with the mitral leaflet behind the regurgitant orifice and impinged to the medial or lateral wall of the left atrium [28]. The adherence and deviation of the jet to the valve leaflets and atrial wall, the so-called Coandǎ effect, causes up to 40% smaller color Doppler jet areas and leads to underestimation of eccentric jets with visual grading [29]. Vena contracta measurements are based on the assumption that the jet has a circular cross-sectional shape and measurements from multiple jets cannot be added [4]. Furthermore, the PISA-method is based on the assumption of hemispheric symmetry of the velocity distribution [4]. Due to these drawbacks, no gold standard is available to quantify the regurgitation of these complex jets with echocardiography and grading of regurgitation remains relatively subjective.

CMR offers non-invasive evaluation of atrial and ventricular dimensions and function with excellent reproducibly [30] within one comprehensive examination without geometric assumptions. Indirect quantification of LAVV regurgitation by subtracting the LAVV forward flow from the aortic flow has been shown feasible in patients with corrected AVSD [31]. Another approach to quantify LAVV regurgitation is the combination of planimetry-derived stroke volume with assessment of aortic flow volume [32]. However, both are indirect assessments of the regurgitant volume, and are not suitable in case of multiple valve lesions, such as aortic valve insufficiency or intra-cardiac shunts, such as a ventricular septal defect. Moreover, planimetry relies on accurate contour definitions, with concomitant intra- and interobserver variability, and separate acquisitions are needed with possible differences in heart rate, possibly explaining the moderate correlation and variation between 4DFlow and the combination of planimetry- and aortic flow. 4DFlow CMR can be used to directly quantify flow over the atrioventricular valves using retrospective valve tracking. This technique has been validated both in vitro and in vivo and proved to be accurate and reproducible [9]. 4DFlow CMR is independent of hemodynamic or geometric assumptions and it has the opportunity to retrospectively position the measurement plane according to flow direction during all phases of the cardiac cycle, and to use separate measurement planes for each jet in case of multiple jets, which is not possible with echocardiography. Furthermore, using 4DFlow CMR the measurement area perpendicular to the regurgitant jet can be adjusted in case of a non-circular cross-section regurgitant jet area, which is not possible using echocardiography. Furthermore, 4DFlow CMR has the advantage of internal validation of flow volume consistency as was shown to be excellent in our study as well as in a recent study in patients with a congenital heart disease [33]. Internal validation with aortic flow was excellent in the current study, including patients with complex jets. However, no gold standard is available that could be used to validate the 4DFlow CMR and therefore, in vivo validation is difficult. Nevertheless, this is the first approach that allows direct quantification of the regurgitant volume without the use of assumptions in this patient group, including children, with challenging LAVV regurgitation jets. The prognostic value of 4DFlow CMR regurgitation quantification has not been studied. Future, long-term follow-up studies are required to investigate cut-off values of quantification of regurgitation with 4DFlow CMR, which might differ from available echocardiography values and how these need to be used in patient management.

In this study, we have to acknowledge some limitations. Although 4DFlow CMR data was acquired, jet angles were measured in 2D planes based on streamline projections. Streamlines represent velocity direction at each location at one instant in time only. By measuring jet angles at different phases of systole, however, we provide useful information on jet direction at specific time points. Analysis of 4DFlow CMR, with manual positioning of the measurement plane, is time-consuming and might be affected by observer variation. However, compared to conventional 2D one-directional velocity-encoded CMR, additional analysis time consists only of time required for the valve tracking, which amounts to approximately 1–2 minutes per valve. Future technical advances may allow full automatic valve tracking and placement of the measurement plane perpendicular to the flow direction, which will reduce analysis time and also eliminate observer variation. Another limitation is that none of the studied patients were classified with severe regurgitation. Although we do not expect different results in these patients, future studies are needed to confirm regurgitant jet characteristics and the feasibility of quantification in patients with severe LAVV regurgitation after AVSD correction.

## Conclusions

4DFlow CMR with streamline visualization revealed multiple, dynamic, non-circular, eccentric regurgitant jets of the LAVV in corrected AVSD patients and enables direct quantification of the regurgitation of these challenging jets. The observed complex jets explicate the limitations of qualitative and quantitative assessment of LAVV in these patients with echocardiography [5,18]. Quantification of LAVV regurgitation with 4DFlow CMR with retrospective valve tracking showed good internal validation and has the potential to be used as an adjunct to echocardiography for more comprehensive evaluation of LAVV regurgitation. Future studies in patient groups with congenital and acquired heart disease are needed to investigate the potential use of 4DFlow in evaluation of mitral valve/LAVV regurgitation and its effect on cardiac function during follow-up.

## References

[1] Hoohenkerk GJ, Bruggemans EF, Rijlaarsdam M, Schoof PH, Koolbergen DR, Hazekamp MG (2010). More than 30 years’ experience with surgical correction of atrioventricular septal defects. Ann Thorac Surg.

[2] Baumgartner H, Bonhoeffer P, De Groot NM, de Haan F, Deanfield JE, Galie N (2010). ESC Guidelines for the management of grown-up congenital heart disease (new version 2010). Eur Heart J.

[3] Biner S, Rafique A, Rafii F, Tolstrup K, Noorani O, Shiota T (2010). Reproducibility of proximal isovelocity surface area, vena contracta, and regurgitant jet area for assessment of mitral regurgitation severity. JACC Cardiovasc Imaging.

[4] Lancellotti P, Moura L, Pierard LA, Agricola E, Popescu BA, Tribouilloy C (2010). European Association of Echocardiography recommendations for the assessment of valvular regurgitation. Part 2: mitral and tricuspid regurgitation (native valve disease). Eur J Echocardiogr.

[5] Prakash A, Lacro RV, Sleeper LA, Minich LL, Colan SD, McCrindle B (2011). Challenges in echocardiographic assessment of mitral regurgitation in children after repair of atrioventricular septal defect. Pediatr Cardiol.

[6] Zoghbi WA, Chambers JB, Dumesnil JG, Foster E, Gottdiener JS, Grayburn PA (2009). Recommendations for evaluation of prosthetic valves with echocardiography and doppler ultrasound: a report from the American Society of Echocardiography’s Guidelines and Standards Committee and the Task Force on Prosthetic Valves, developed in conjunction with the American College of Cardiology Cardiovascular Imaging Committee, Cardiac Imaging Committee of the American Heart Association, the European Association of Echocardiography, a registered branch of the European Society of Cardiology, the Japanese Society of Echocardiography and the Canadian Society of Echocardiography, endorsed by the American College of Cardiology Foundation, American Heart Association, European Association of Echocardiography, a registered branch of the European Society of Cardiology, the Japanese Society of Echocardiography, and Canadian Society of Echocardiography. J Am Soc Echocardiogr.

[7] Thavendiranathan P, Phelan D, Thomas JD, Flamm SD, Marwick TH (2012). Quantitative assessment of mitral regurgitation: validation of new methods. J Am Coll Cardiol.

[8] Roes SD, Hammer S, van der Geest RJ, Marsan NA, Bax JJ, Lamb HJ (2009). Flow assessment through four heart valves simultaneously using 3-dimensional 3-directional velocity-encoded magnetic resonance imaging with retrospective valve tracking in healthy volunteers and patients with valvular regurgitation. Invest Radiol.

[9] Westenberg JJ, Roes SD, Ajmone MN, Binnendijk NM, Doornbos J, Bax JJ (2008). Mitral valve and tricuspid valve blood flow: accurate quantification with 3D velocity-encoded MR imaging with retrospective valve tracking. Radiology.

[10] Calkoen EE, Roest AA, Kroft LJ, van der Geest RJ, Jongbloed MR, van den Boogaard PJ, et al. Characterization and improved quantification of left ventricular inflow using streamline visualization with 4DFlow MRI in healthy controls and patients after atrioventricular septal defect correction. J Magn Reson Imaging. 2014; doi: 10.1002/jmri.24735. 10.1002/jmri.24735 25143314

[11] Du BD, Du Bois EF (1989). A formula to estimate the approximate surface area if height and weight be known. 1916. Nutrition.

[12] Napel S, Lee DH, Frayne R, Rutt BK (1992). Visualizing three-dimensional flow with simulated streamlines and three-dimensional phase-contrast MR imaging. J Magn Reson Imaging.

[13] Wolf RL, Ehman RL, Riederer SJ, Rossman PJ (1993). Analysis of systematic and random error in MR volumetric flow measurements. Magn Reson Med.

[14] Kayser HW, Stoel BC, van der Wall EE, van der Geest RJ, de Roos A (1997). MR velocity mapping of tricuspid flow: correction for through-plane motion. J Magn Reson Imaging.

[15] Lotz J, Meier C, Leppert A, Galanski M (2002). Cardiovascular flow measurement with phase-contrast MR imaging: basic facts and implementation. Radiographics.

[16] Bland JM, Altman DG (1986). Statistical methods for assessing agreement between two methods of clinical measurement. Lancet.

[17] Takahashi K, Mackie AS, Thompson R, Al-Naami G, Inage A, Rebeyka IM (2012). Quantitative real-time three-dimensional echocardiography provides new insight into the mechanisms of mitral valve regurgitation post-repair of atrioventricular septal defect. J Am Soc Echocardiogr.

[18] Li JS, Colan SD, Sleeper LA, Newburger JW, Pemberton VL, Atz AM (2011). Lessons learned from a pediatric clinical trial: the Pediatric Heart Network angiotensin-converting enzyme inhibition in mitral regurgitation study. Am Heart J.

[19] Ling LH, Enriquez-Sarano M, Seward JB, Tajik AJ, Schaff HV, Bailey KR (1996). Clinical outcome of mitral regurgitation due to flail leaflet. N Engl J Med.

[20] Enriquez-Sarano M, Avierinos JF, Messika-Zeitoun D, Detaint D, Capps M, Nkomo V (2005). Quantitative determinants of the outcome of asymptomatic mitral regurgitation. N Engl J Med.

[21] Delahaye JP, Gare JP, Viguier E, Delahaye F, De GG, Milon H (1991). Natural history of severe mitral regurgitation. Eur Heart J.

[22] Avierinos JF, Gersh BJ, Melton LJ, Bailey KR, Shub C, Nishimura RA (2002). Natural history of asymptomatic mitral valve prolapse in the community. Circulation.

[23] Bonow RO, Carabello BA, Chatterjee K, de Leon ACJ, Faxon DP, Freed MD (2008). 2008 focused update incorporated into the ACC/AHA 2006 guidelines for the management of patients with valvular heart disease: a report of the American College of Cardiology/American Heart Association Task Force on Practice Guidelines. J Am Coll Cardiol.

[24] Carabello BA, Nolan SP, McGuire LB (1981). Assessment of preoperative left ventricular function in patients with mitral regurgitation: value of the end-systolic wall stress-end-systolic volume ratio. Circulation.

[25] Rusinaru D, Tribouilloy C, Grigioni F, Avierinos JF, Suri RM, Barbieri A (2011). Left atrial size is a potent predictor of mortality in mitral regurgitation due to flail leaflets: results from a large international multicenter study. Circ Cardiovasc Imaging.

[26] Grigioni F, Avierinos JF, Ling LH, Scott CG, Bailey KR, Tajik AJ (2002). Atrial fibrillation complicating the course of degenerative mitral regurgitation: determinants and long-term outcome. J Am Coll Cardiol.

[27] Bonow RO (2014). Left atrial function in mitral regurgitation: guilt by association. JACC Cardiovasc Imaging.

[28] Hall SA, Brickner ME, Willett DL, Irani WN, Afridi I, Grayburn PA (1997). Assessment of mitral regurgitation severity by Doppler color flow mapping of the vena contracta. Circulation.

[29] Ginghina C (2007). The Coanda effect in cardiology. J Cardiovasc Med (Hagerstown).

[30] Grothues F, Smith GC, Moon JC, Bellenger NG, Collins P, Klein HU (2002). Comparison of interstudy reproducibility of cardiovascular magnetic resonance with two-dimensional echocardiography in normal subjects and in patients with heart failure or left ventricular hypertrophy. Am J Cardiol.

[31] Fujita N, Chazouilleres AF, Hartiala JJ, O'Sullivan M, Heidenreich P, Kaplan JD (1994). Quantification of mitral regurgitation by velocity-encoded cine nuclear magnetic resonance imaging. J Am Coll Cardiol.

[32] Kizilbash AM, Hundley WG, Willett DL, Franco F, Peshock RM, Grayburn PA: Comparison of quantitative Doppler with magnetic resonance imaging for assessment of the severity of mitral regurgitation. Am.J.Cardiol. 1998;81:792-795. 10.1016/s0002-9149(97)01024-2 9527098

[33] Hsiao A, Tariq U, Alley MT, Lustig M, Vasanawala SS. Inlet and outlet valve flow and regurgitant volume may be directly and reliably quantified with accelerated, volumetric phase-contrast MRI. J Magn Reson Imaging. 2014. doi:10.1002/jmri.24578. 10.1002/jmri.24578 PMC4126899 24677253

